# Protocol for a systematic review of psychological interventions for cancer-related fatigue in post-treatment cancer survivors

**DOI:** 10.1186/s13643-015-0160-x

**Published:** 2015-12-04

**Authors:** Teresa Corbett, Declan Devane, Jane C. Walsh, AnnMarie Groarke, Brian E. McGuire

**Affiliations:** School of Psychology, National University of Ireland Galway, Galway, Ireland; School of Nursing and Midwifery, National University of Ireland Galway, Galway, Ireland

**Keywords:** Survivorship, Post-treatment, Tiredness, Lethargy, Psychosocial, Therapy, Trials, Low energy, Malignancy, Tumour, Patients, Re-entry period

## Abstract

**Background:**

Fatigue is a common symptom in cancer patients that can persist beyond the curative treatment phase. Some evidence has been reported for interventions for fatigue during active treatment. However, to date, there is no systematic review on psychological interventions for fatigue after the completion of curative treatment for cancer. This is a protocol for a systematic review that aims to evaluate the effectiveness of psychological interventions for cancer-related fatigue in post-treatment cancer survivors. This systematic review protocol was registered with the International Prospective Register of Systematic Reviews (PROSPERO) database.

**Methods/design:**

We will search the Cochrane Central Register of Controlled Trials (CENTRAL; The Cochrane Library), PubMed, MEDLINE, EMBASE, CINAHL, PsycINFO, and relevant sources of grey literature. Randomised controlled trials (RCTs) which have evaluated psychological interventions in adult cancer patients after the completion of treatment, with fatigue as an outcome measure, will be included. Two review authors will independently extract data from the selected studies and assess the methodological quality using the Cochrane Collaboration Risk of Bias Tool.

**Discussion:**

Most existing evidence on cancer-related fatigue is from those in active cancer treatment. This systematic review and meta-analysis will build upon previous evaluations of psychological interventions in people during and after cancer treatment. With the growing need for stage-specific research in cancer, this review seeks to highlight a gap in current practice and to strengthen the evidence base of randomised controlled trials in the area.

**Systematic review registration:**

PROSPERO CRD42014015219.

## Background

### Description of the condition

Cancer patients are often confronted with lingering physical, psychological, and interpersonal challenges that extend into longer term survivorship [1]. These individuals must face disease-specific concerns such as fear of cancer recurrence, concerns regarding body image and sexuality, and financial burdens [2]. As the number of survivors of cancer continues to increase, identification of the best methods for promoting the well-being of long-term survivors is essential [1]. Survivors report that cancer-related fatigue (CrF) is a persistent and distressing symptom in the months and years after the completion of successful treatment of cancer [3].

The National Comprehensive Cancer Network (NCCN) [4] defined CrF as “a distressing persistent sense of tiredness or exhaustion related to cancer that is not proportional to recent activity and interferes with usual functioning” [4]. Koornstra et al. [5] described CrF as one of the most common symptoms experienced by oncology patients. Despite this, most patients do not expect long-term fatigue after treatment and are not routinely warned of the possibility of such persistent symptoms. Depending on the measurement criteria used, rates of reported post-treatment CrF range from 17 to 53 %. About one fifth of cancer survivors report persistent, severe fatigue in the first year following anti-cancer therapy [5]. Bower [6] reported that approximately 30 % of patients experience persistent fatigue that may endure for 10 years or more. In this review, the term “survivor” will refer to anyone after the completion of successful treatment of cancer.

Minton et al. [3] describe post-treatment CrF as a sensation ranging from tiredness to exhaustion that impacts all aspects of quality of life. The symptoms affect the individual’s physical, emotional, social and/or cognitive well-being. Fatigue limits the ability to function, socialise, and participate in previously enjoyable activities and, ultimately, the individual’s capacity to lead a normal life [5]. Patients frequently describe fatigue as having a greater negative impact on their daily lives than many other cancer- or treatment-related complications. Fatigue has also been linked to shorter recurrence-free and overall survival rates in patients with cancer [6]. Despite its deleterious impact on patient quality of life, CrF often remains unrecognised and untreated [5]. CrF is an abstract, multifaceted problem, with many potential etiologies [7]. These include alterations in sleep cycles, physical and emotional pain, cytokine release, immune dysfunction, premorbid functioning and illnesses, anxiety, and the product of certain coping mechanisms. CrF is recognised as a long-term consequence of treatment by the UK National Health Service and by the European Organisation for Research and Treatment of Cancer (EORTC). Koornstra et al. [5] indicate that the management of CrF is currently suboptimal.

### Description of the intervention

Psychological interventions for CrF can have multiple components, such as education regarding cancer and its treatment, provision of emotional support, and training in coping skills such as challenging unhelpful thoughts and relaxation training. Research that focuses on psychological interventions has demonstrated improvements in fatigue [8]. These interventions have included psychological, educational, and support group studies, supportive therapies, and psycho-education [3]. Psychological therapies may change the way a person interprets their fatigue, and how the individual responds to symptoms and manages them, by promoting flexibility in thinking and by enabling ongoing involvement in everyday activities [9].

### How the intervention might work

Psychological treatments for fatigue may also work by reducing psychological arousal through the use of relaxation training. Further, psychological interventions may aim to address mood and goal-setting, self-efficacy, and incremental goal attainment [10].

Koornstra et al. [5] identified psychological strategies that could be useful for individuals aiming to reduce CrF. Energy conservation and daily self-monitoring of fatigue levels can help to establish a routine, balancing activity and rest. Behavioural and sleep hygiene techniques can be recommended for improving sleep patterns. Teaching stress management techniques and relaxation techniques may improve sleep and decrease distress/anxiety. Cognitive behavioural therapy has been associated with managing fatigue symptoms in heterogeneous samples of patients undergoing medical treatments for cancer [11].

### Why it is important to do this review

Smedslund and Ringdal [12] propose that the best method of studying associations between psychological factors and cancer is the controlled intervention study. However, few psychological controlled interventions have been directed toward cancer survivors beyond the early diagnostic and treatment phase, so that long-term survivors have not received due attention [3]. It is still unclear how effective such interventions may be in post-treatment cancer patients during the remission or re-entry stages. Structured rehabilitation has been found to result in sustained improvements in fatigue, particularly in patients who have completed treatment and are in the survivorship phase.

Johnson et al. [13] highlighted that interventions informed by theory are more effective than interventions lacking a theoretical basis. Stanton [1] reported that interventions explicitly designed to enhance the capacity to monitor and alter cancer-relevant thoughts, emotions, and behaviours produce larger effect sizes than do interventions lacking those components. Studies that explicitly incorporated psychological processes such as self-efficacy, mastery, optimism, and learned resourcefulness have been reported to influence CrF levels [5].

Many psychological interventions have been evaluated with patients with CrF but are often without a specific focus on fatigue within the treatment programme. Interventions that lack a specific focus on fatigue have rarely been found to be effective in reducing fatigue [14].

Findings are mixed in their conclusions regarding the effectiveness of psychological interventions for CrF [1]. Some interventions have reported strong positive effects, whereas others produced negative findings. Bower [6] presented evidence that psychological and integrative medicine approaches may have beneficial effects on persistent post-treatment fatigue, but preliminary conclusions are limited by small sample sizes in several trials.

### Objective

The objective of this study is to evaluate the effectiveness of psychological interventions for cancer-related fatigue in post-treatment cancer survivors.

## Methods

### Criteria for considering studies for this review

This systematic review and meta-analysis will be conducted and reported in accordance with the Preferred Reporting Items for Systematic Reviews and Meta-Analyses (PRISMA) statement [15]. This systematic review protocol was registered with the International Prospective Register of Systematic Reviews (PROSPERO) database (registration number: CRD42014015219).

The PRISMA checklist [15] encourages authors to describe eligibility criteria using the PICO reporting system (which describes the participants, interventions, comparisons, outcome(s), and study design of the included studies).

### Types of studies

This review will include randomised controlled trials (RCTs) that compare credible psychological treatments with treatment as usual or waiting list control. Studies will be included regardless of treatment intensity or duration, mode of treatment delivery (e.g. individual, group), or medium of treatment (e.g. in-person, online).

### Types of participants

Studies involving adults 18 years and older who have completed treatment for cancer will be included regardless of gender, tumour type, and type of treatment. Survivorship of cancer or “survivorship” has been defined in various ways. One definition describes the process of living with, through, and beyond cancer, with cancer survivorship beginning at diagnosis. Another definition defines survivorship as the period after the completion of treatment when the patient has no disease [16]. In this review, the term survivorship will refer to the latter definition.

### Types of interventions

Studies must evaluate the effect of psychological treatments designed to manage CrF. Studies reporting the effects of interventions on multiple outcomes will be included if fatigue is one of the outcomes of interest. Interventions including psychotherapy and psycho-education will be included in this study. Other interventions that will be considered for inclusion will focus on cognitive restructuring and coping strategies. Educational interventions are those that provide advice or information (verbal, written, audio-visual, or computer-delivered material) with the aim of helping people understand and manage CrF. Studies that use methods such as meditation, relaxation, or techniques to improve coping with fatigue will also be included. Cognitive behavioural therapy, self-help or self-care, relaxation, or stress management will be included. In line with McGuire et al. [9], studies that combine psycho-behavioural and non-psychological methods will be included if there is a predominant emphasis on a psychological element in the study design. Studies will be excluded if they do not employ a credible psychotherapeutic rationale or theory in the intervention design.

The following comparisons will be assessed:

1. Psychological interventions (all types) vs usual care
2. Subgroups of specific psychological intervention type (e.g. cognitive behavioural therapy) vs usual care

(See section on “Subgroup analysis and investigation of heterogeneity” for further information on comparisons.)

### Types of outcome measures

#### Primary outcomes

Included studies must have fatigue as an outcome of interest. This will incorporate fatigue measured as a main outcome or within a cluster measurement of physical symptoms or quality of life.

In line with Goedendorp et al. [14], studies will be included if fatigue was measured with a questionnaire specifically designed to evaluate fatigue. Fatigue subscales as part of a broader quality-of-life measure will be included, provided that specific fatigue-related data are available. Fatigue may also be measured with a visual analogue scale (VAS) or as part of a symptom list and scored as “present” or “absent”.

Fatigue may be measured in terms of characteristics such as intensity, distress, duration, or frequency, or as dimensions such as physical fatigue, mental fatigue, or general fatigue.

#### Secondary outcomes

Secondary outcomes will include functional impact of fatigue (self-report questionnaire measures assessing the impact of fatigue on daily functioning), fatigue self-efficacy (self-reported scales of control or self-efficacy in relation to fatigue), mood (self-reported scales of depression, and/or anxiety, or distress), and global quality of life (self-report questionnaire measures assessing the impact of fatigue on quality of life).

### Search methods for identification of studies

No date restriction will be imposed on the studies. Studies will be included if a full-text paper in English is made available, either through databases or through contact with the study authors. Where available, protocol methods will be compared with the methods and results reported in the included study.

### Electronic searches

We will search the following electronic databases: Cochrane Central Register of Controlled Trials (CENTRAL), MEDLINE, EMBASE, CINAHL, PsycINFO, Web of Science, and CancerLit. The same search strategies will be used with alterations as appropriate for each database interface. Details of the search strategy are provided in Table 1. We will use Medical Subject Headings (MeSH) or equivalent and text word terms.

**Table 1:** Details of the search strategy

	Search term
1	(“cancer survivors” or neoplasm or survivor or cancer or remission)
2	(Psychology or Psychotherapy or Behaviour Therapy or hypnosis or relaxation or imagery or cognition or psychotherapy or cognitive)
3	(fatigue or asthenia or asthenic or asthenia or (exhaustion or exhausted) or “loss of energy” or “loss of vitality” or (weary or weariness or weakness) or (apathy or apathetic or lassitude or lethargic or lethargy) or (sleepy or sleepiness or drowsy or drowsiness) or (tired or tiredness))
4	(randomized controlled trial or controlled clinical trial or “random assignment”)

### Searching other resources

Unpublished and ongoing trials will be identified by checking trials and protocols published on relevant databases of current ongoing clinical research studies (e.g. https://clinicaltrials.gov). Trial registries and conference abstracts will also be searched. The lead or contact authors of all identified studies will be asked to identify further studies where possible. Grey literature will be searched using the OpenGrey database (www.opengrey.eu/), which includes technical or research reports, doctoral dissertations, and conference papers from the previous 5 years (e.g. from annual American Society of Clinical Oncology (ASCO) or International Psycho-Oncology Society World Congress (IPOS) conferences). Further published, unpublished and ongoing trials will be identified by checking trials and protocols published on the following clinical trial registers and websites:

- World Health Organization International Clinical Trials Registry Platform (WHO ICTRP; www.who.int/ictrp/en)
- metaRegister of Controlled Trials (mRCT; http://www.controlled-trials.com/)
- ClinicalTrials.gov (www.clinicaltrials.gov)
- www.cancer.gov/clinicaltrials

### Data collection and analysis

#### Selection of studies

One review author (TC) will initially screen titles and abstracts and eliminate those obviously not relevant to this review. Two review authors (TC and BMG) will independently screen the remaining titles and abstracts for their eligibility for inclusion in accordance with the above defined criteria. Ineligible studies will be excluded at this stage, and the authors will record the reason for rejection. When the title and abstract do not provide all the information concerning the criteria, full paper copies will be retrieved and screened. We will retrieve full-text copies of all studies if either review author determines that the study possibly or definitely meets the inclusion criteria.

Disagreements between the two reviewers will be resolved by discussion, with the involvement of a third reviewer where agreement cannot be reached (DD, JW, or AMG). Multiple reports of the same study will be counted as a single study. The PRISMA template will be used to produce a flow chart showing details of studies included and excluded at each stage of the study selection process.

#### Data extraction and management

Two review authors (TC and BMG) will independently extract data from the studies using a specifically designed data extraction form. The form will be piloted on a sample of three studies and then altered if required before full data extraction begins. Discrepancies will be resolved by discussion, with the involvement of a third reviewer where necessary. Authors will be contacted in order to obtain any missing data. Findings will be reported regardless of their direction. Positive and negative findings must be clearly defined in the included studies. The following information will be extracted from the studies:

- Participant characteristics including demographic characteristics (e.g. age, gender).
- Disease-specific factors such as cancer type/stage and type of treatment.
- Minimum time since completion of treatment.
- Geographic location of study.
- Psychological technique/therapy.
- Intervention information for each arm of the study (type of delivery, content, duration, treatment dose received by the participants, comparison/s).
- Descriptions of providers of the intervention and comparison intervention/s.
- Timing of assessment for each outcome.
- Adverse events reported by studies will be addressed. However, given that data on adverse effects is often not provided, the absence of information will not be inferred to imply that the intervention was entirely safe. Similarly, studies in which adverse effects are carefully sought will not necessarily be deemed unsafe. This is due to the fact that adverse events will occur with higher frequency in such papers than in studies in which they are sought less carefully or left unreported [17]. If any information is missing or unclear, clarification will be ascertained through personal contact with the investigators.

#### Assessment of risk of bias in included studies

Criteria for features of the RCT design are based on those set out by the Cochrane Collaboration Risk of Bias Tool and will be considered for each of the included studies in order to assess risk of bias. These criteria include the following:

- Random sequence generation: To check for selection bias (biased allocation to interventions) due to inadequate generation of a randomised sequence.
- Allocation concealment: To check for selection bias (biased allocation to interventions) due to inadequate concealment of allocations prior to assignment.
- Performance bias: To check for bias due to knowledge of the allocated interventions by participants and personnel during the study.
- Detection bias: To check for bias due to knowledge of the allocated interventions by outcome assessors.
- Attrition bias: To check for bias due to amount, nature, or handling of incomplete outcome data.
- Selective reporting bias: To check for bias due to selective outcome reporting by comparing in-publication reporting of the outcomes of interest reported in the methods section to those reported in the results section. As with Lutomski et al. [18], high risk would be reported where pre-specified outcomes were not all reported, any primary outcomes were not pre-specified, and reporting of key outcomes were incomplete.
- Other sources of bias considered to evaluate the quality of the intervention will include potential bias due to baseline differences, inappropriate influence of the study sponsor, and early stopping for benefit [19].

Each domain will be judged independently by two authors as high, low, or unclear risk of bias. Discrepancies will be resolved by discussion, with the involvement of a third reviewer where necessary.

#### Measures of treatment effect

We will use The Cochrane Collaboration’s Review Manager Software, RevMan 5, for all analyses. For continuous data, we will report the mean differences between groups and the 95 % confidence interval (95 % CI). Where no standard deviations are reported, we will calculate the standard deviation using the methods described in the Cochrane Handbook for Systematic Reviews of Interventions [20]. Where the same outcome is measured using different measurement tools, we will calculate the standardised mean difference and the 95 % CI for continuous data.

#### Assessment of heterogeneity

Statistical heterogeneity will be tested using *χ*^2^, *I*^2^, and *Τ*^2^. Statistical heterogeneity will be regarded as substantial where *Τ*^2^ is greater than 0.00 and the *χ*^2^*p* value is <0.1 or *I*^2^ is >50 %. Data will be analysed using RevMan 5.

#### Assessment of reporting biases

As with Bourke et al. [21], we will examine funnel plots corresponding to meta-analysis of the primary outcomes to assess the potential for small-study effects such as publication bias if a sufficient number of studies (i.e. more than 10) are identified.

#### Data synthesis

Data will only be pooled if it is clinically meaningful and appropriate to do so. Otherwise, a narrative synthesis of the data will be conducted.

Continuous data will be combined only where (i) means and standard deviations are available or calculable and (ii) there is no clear evidence of skew in the distribution (using methods described in the Cochrane Handbook for Systematic Reviews of Interventions (2011)).

If it is possible to combine mean differences of scales measuring the same clinical outcomes in different ways, they will be standardised in order to combine results across scales (otherwise, weighted mean differences will be used).

We think it likely that there will be clinical heterogeneity driven by differences in interventions, comparators, and participants sufficient to expect that the underlying treatment effects would differ between the included trials. We will therefore use a random-effects meta-analysis model to produce an overall summary of the average treatment effect across included trials.

#### Subgroup analysis and investigation of heterogeneity

We hypothesise that each of the factors below has the potential to have a clinically meaningful effect on the response to a psychological intervention among fatigued post-treatment cancer survivors. Therefore, if sufficient data are available, we will undertake subgroup analysis based on the following:

1. Psychological intervention type A vs psychological intervention type B
  If sufficient evidence is available, in order to establish if there is a superior method for treating fatigue in cancer survivors, differences between types of therapies will be explored (e.g. cognitive behavioural therapy, mindfulness therapy).
2. Intervention for specific cancer type only vs intervention for any cancer type
  Goedendorp et al. [14] noted that many of the studies in their review of fatigued cancer patients during cancer were carried out with only breast cancer patients. However, they found that the studies were too heterogeneous to draw additional conclusions about interventions for specific groups. In this review, if possible, interventions designed for specific patient groups will be compared to those interventions that include all (or more than one) cancer type.
3. In-person interventions vs remote interventions
  Where possible, studies will be compared to establish if sessions should be based on face-to-face contact or if remote interventions online or with telephone sessions might be an alternative.
4. Interventions specifically designed to treat fatigue after cancer treatment vs interventions not specific for fatigue
  Goedendorp et al. [14] found that the effectiveness of interventions specific for fatigue was significantly higher than interventions not specific for fatigue. Therefore, this review will compare interventions that were specifically designed to treat fatigue after cancer treatment and interventions not specific for fatigue.

### Sensitivity analysis

#### Trial quality

1. We will conduct a sensitivity analysis based on trial quality, whereby studies of high or unclear risk of bias across different domains (see section on “Assessment of risk of bias in included studies”) will be excluded in order to assess for any substantive difference to the overall effect estimates. If no substantive difference exists, the studies will be left in for the main analysis. This sensitivity analysis will be conducted for the primary outcomes only.

#### Outcome validity

1. Given that there is no accepted definition of cancer-related fatigue (CrF) and no agreement on how it should be measured [22], a sensitivity analysis will be conducted based on outcome measurement. Scales may vary in the quality of psychometric properties. Studies that confirm that the scales are validated measures of fatigue will be compared to those that do not meet the following a priori criteria, based on criteria outlined by Minton and Stone [22].
  The paper that used the scale should have referred to at least three of the following: internal consistency, test–retest reliability, known group validity (discriminant validity), responsiveness to change, or convergent validity (against other scales). The original scale reference will be accessed if this information is not provided. The original paper will also be cross-referenced for citing articles to assess the frequency of the scale use and the type of populations studied.
2. Further, single-parameter fatigue subscales as part of a broader quality-of-life measure will be compared to multiple-parameter scales that were specifically designed to assess fatigue.

## Discussion

This proposed review will add to the literature in several ways. Due to advancements in cancer treatments, more efficient and effective screening programmes, and an aging population, there has been a significant increase in the incidence of cancer and the life expectancy of cancer patients [23]. Cancer survivorship issues, such as the long-term side effects of cancer treatment and their impact on quality of life (QOL), are becoming increasingly pertinent.

Due to the multifactorial and complex nature of CrF, the pathophysiology is not well understood. There are currently no proven pharmacologic treatments for CrF, and high-level evidence to guide coping with CrF is limited [24]. CrF appears to be elicited during the treatment phase, but Gielissen et al. [25] reported that there is no clear relationship between the initial cancer, treatment variables, and persistent fatigue. There is no recommended strategy for managing fatigue in cancer survivors. It may be the case that cancer itself and/or cancer treatment triggered fatigue, but other factors could be responsible for persistence of fatigue complaints. These include psychological and behavioural factors such as physical activity, sleep quality, and fear of disease recurrence [25].There is mounting evidence that cognitive and emotional factors play pivotal roles in the experience of cancer-related fatigue.

The majority of trials on CrF are conducted during treatment and are rarely tested or tailored to those who have concluded active cancer treatment [5]. Minton et al. [3] suggest that this lack of recognition may in part be linked to a lack of knowledge relating to the efficacy of interventions for fatigue. It is necessary to identify for whom and under what conditions psychological interventions are most effective [8].

There are a few potential limitations of this planned review. Clinical expertise and preliminary review of some of the relevant studies for this research show that there are many issues in defining and measuring fatigue, due to its subjective nature. We will address this by conducting a sensitivity analysis based on those studies that use standardised and validated measures of fatigue. Studies may be lacking complete information (e.g. severity of patients’ condition, time since treatment, co-morbidities). While it will be feasible to capture and comment on the extent of information collected and adjusted for, the impact of any missing information or comparisons will be difficult to assess.

As the majority of existing evidence on CrF is from those in active cancer treatment, the results of this systematic review and meta-analyses will be of interest to patients post cancer treatment, policymakers, researchers, and clinicians working in survivorship health care. With the growing need for stage-specific research in cancer, this review seeks to highlight a gap in current practice and to strengthen the evidence base of randomised controlled trials in the area. To our knowledge, no previous review has examined the effectiveness of psychological interventions for those with persistent CrF after the completion of curative treatment.

## Abbreviations

AMG: AnnMarie Groarke
ASCO: American Society of Clinical Oncology
BMG: Brian E. McGuire
CrF: cancer-related fatigue
DD: Declan Devane
EORTC: European Organisation for Research and Treatment of Cancer
IPOS: International Psycho-Oncology Society World Congress
JW: Jane Walsh
MeSH: Medical Subject Headings
mRCT: metaRegister of Controlled Trials
NCCN: National Comprehensive Cancer Network
PICO: Participants, Interventions, Comparisons, Outcome(s)
PRISMA: Preferred Reporting Items for Systematic Reviews and Meta-Analyses
QOL: quality of life
RCT: randomised controlled trial
TC: Teresa Corbett
WHO ICTRP: World Health Organization International Clinical Trials Registry Platform
VAS: visual analogue scale
